# A 1:2 co-crystal of 2,2′-di­thiodi­benzoic acid and benzoic acid: crystal structure, Hirshfeld surface analysis and computational study

**DOI:** 10.1107/S2056989018017097

**Published:** 2019-01-01

**Authors:** Sang Loon Tan, Edward R. T. Tiekink

**Affiliations:** a Research Centre for Crystalline Materials, School of Science and Technology, Sunway University, 47500 Bandar Sunway, Selangor Darul Ehsan, Malaysia

**Keywords:** crystal structure, di­thiodi­benzoic acid, benzoic acid, hydrogen bonding, computational chemistry

## Abstract

The asymmetric unit of the title 1:2 co-crystal, C_14_H_10_O_4_S_2_·2C_7_H_6_O_2_, comprises half a mol­ecule of di­thiodi­benzoic acid [systematic name: 2-[(2-carb­oxy­phen­yl)disulfan­yl]benzoic acid, DTBA], as the mol­ecule is located about a twofold axis of symmetry, and a mol­ecule of benzoic acid (BA). The DTBA mol­ecule is twisted about the di­sulfide bond [the C—S—S—C torsion angle is −83.19 (8)°] resulting in a near perpendicular relationship between the benzene rings [dihedral angle = 71.19 (4)°]. The carb­oxy­lic acid group is almost co-planar with the benzene ring to which it is bonded [dihedral angle = 4.82 (12)°]. A similar near co-planar relationship pertains for the BA mol­ecule [dihedral angle = 3.65 (15)°]. Three-mol­ecule aggregates are formed in the crystal whereby two BA mol­ecules are connected to a DTBA mol­ecule via hy­droxy-O—H⋯O(hydroxy) hydrogen bonds and eight-membered {⋯HOC=O}_2_ synthons. These are connected into a supra­molecular layer in the *ab* plane through C—H⋯O inter­actions. The inter­actions between layers to consolidate the three-dimensional architecture are π–π stacking inter­actions between DTBA and BA rings [inter-centroid separation = 3.8093 (10) Å] and parallel DTBA-hy­droxy-O⋯π(BA) contacts [O⋯ring centroid separation = 3.9049 (14) Å]. The importance of the specified inter­actions as well as other weaker contacts, *e.g.* π–π and C—H⋯S, are indicated in the analysis of the calculated Hirshfeld surface and inter­action energies.

## Abstract 2

The asymmetric unit of the title 1:2 co-crystal has a half mol­ecule of twofold symmetric di­thiodi­benzoic acid and a full mol­ecule of benzoic acid. These are connected into three-mol­ecule aggregates via hy­droxy-O—H⋯O(hy­droxy) hydrogen bonds.

## Chemical context   

Mol­ecular recognition represents an essential aspect in the crystal engineering of co-crystals as it dictates how supra­molecular aggregates are formed, whether through shape, size or functional complementarity, to give a distinct connectivity and pattern (Meng *et al.*, 2008 ▸). To date, various supra­molecular frameworks comprising homo-synthons, occurring between the same functional groups, as well as hetero-synthons, occurring between disparate functional groups, have been described. Mol­ecules with carb­oxy­lic acid functionality remain at the forefront of co-crystal technology based on hydrogen-bonded synthons (Duggirala *et al.*, 2015 ▸). Despite expectations to the contrary, the carb­oxy­lic acid⋯carb­oxy­lic acid homo-synthon, *i.e*. association through the formation of an eight-membered {⋯HOC=O}_2_ synthon, only forms in about one-third of structures where they potentially can occur (Allen *et al.*, 1999 ▸). The remaining structures of carb­oxy­lic acids are dominated by hetero-synthons involving carb­oxy­lic acid with other functional groups, such as a pyridyl residue (Shattock *et al.*, 2008 ▸). This relatively low probability is due to competing supra­molecular inter­actions that hinder the formation of the homosynthon (Steiner, 2001 ▸). A related issue concerns the formation of co-crystals involving different carb­oxy­lic acids (Seaton, 2011 ▸). Here, different crystalline outcomes may be envisaged and in terms of co-crystals, co-crystals involving the same mol­ecules associating via a symmetric carb­oxy­lic acid homosynthon might be isolated, or a co-crystal comprising different mol­ecules, via a non-symmetric homo-synthon might be formed. In this context, in a recent study, the characterization of the 2:1 co-crystal between 2,2′-di­thiodi­benzoic acid (DTBA) and 3-chloro­benzoic acid showed the formation of a homo-synthon between two DTBA mol­ecules with each of the terminal carb­oxy­lic acid residues of the two-mol­ecule aggregate engaged in non-symmetric homo-synthons with two 3-chloro­benzoic acid mol­ecules, giving rise to a hydrogen-bonded four-mol­ecule aggregation pattern (Tan & Tiekink, 2019 ▸). In continuation of these studies, herein, the crystal and mol­ecular structures of the title 1:2 co-crystal of DTBA and benzoic acid (BA) are described as well as an analysis of the calculated Hirshfeld surface and the calculation of some specific inter­action energies through a computational chemistry approach.
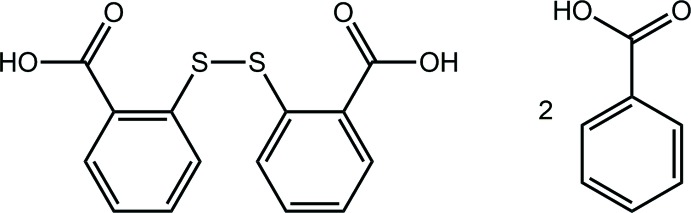


## Structural commentary   

**Figure 1:**
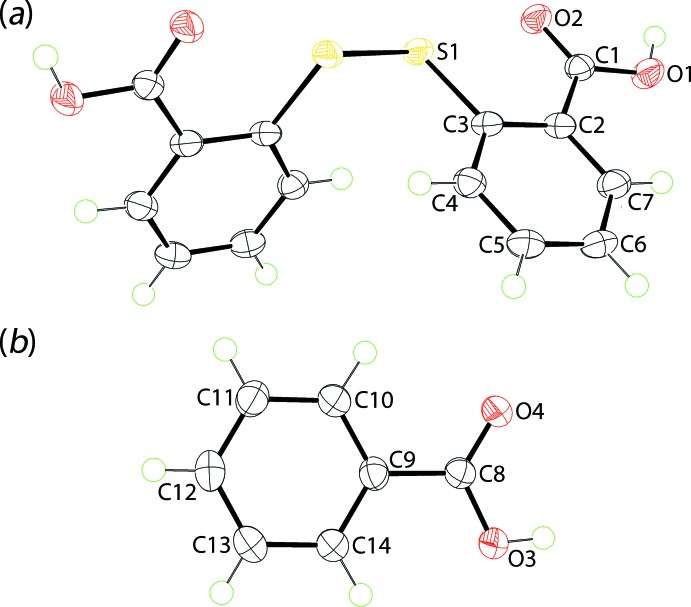
The mol­ecular structures of (*a*) 2,2′-di­thiodi­benzoic acid and (*b*) benzoic acid in (I), showing the atom-labelling scheme and displacement ellipsoids at the 70% probability level. The mol­ecule in (*a*) is disposed about a twofold axis of symmetry with unlabelled atoms related by the symmetry operation: 1 − *x*, *y*, 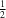 − *z*.

The title co-crystal (I) was the result of crystallization of a powder resulting from the solvent-assisted (methanol) grinding of a 1:1 mixture of 2-thio­benzoic acid and benzoic acid. X-ray crystallography showed the asymmetric unit of the resultant crystals to comprise half a mol­ecule of 2,2′-di­thiodi­benzoic acid (DTBA), as this is disposed about a crystallographic twofold axis of symmetry, Fig. 1 ▸(*a*), and a mol­ecule of benzoic acid (BA) in a general position, Fig. 1 ▸(*b*). Such oxidation of the original 2-thio­benzoic acid to DTBA is well known in co-crystallization studies (Broker & Tiekink, 2007 ▸; Gorobet *et al.*, 2018 ▸). In terms of stoichiometry, the formation of the title 1:2 co-crystal is consistent with the 1:1 stoichiometry of the original grinding experiment.

The twofold-symmetric DTBA mol­ecule is twisted about the di­sulfide bond with the C3—S1—S1^i^—C3^i^ torsion angle being −83.19 (8)°; symmetry operation (i): 1 − *x*, *y*, 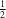 − *z.* This almost orthogonal disposition is also seen in the dihedral angle between the benzene rings of 71.19 (4)°. The presence of a carb­oxy­lic acid group is readily confirmed by the disparity in the C1—O1, O2 bond lengths, *i.e*. 1.317 (2) and 1.229 (2) Å, respectively. This group is practically co-planar with the benzene ring to which it is bonded, as seen in the dihedral angle of 4.82 (12)°. This co-planar arrangement allows for a significant intra­molecular S←O inter­action, *i.e*. S1⋯O2 = 2.6712 (12) Å, as the carbonyl-O2 atom is orientated towards a di­sulfide-S1 atom (Nakanishi *et al.*, 2007 ▸).

The presence of a carb­oxy­lic acid group in the mol­ecule of BA is confirmed by the C8—O3, O4 bond lengths of 1.318 (2) and 1.233 (2) Å, respectively. As for the DTBA mol­ecule, the carb­oxy­lic acid group is close to co-planar with the benzene ring to which it is bound, forming a dihedral angle of 3.65 (15)°.

## Supra­molecular features   

**Figure 2:**
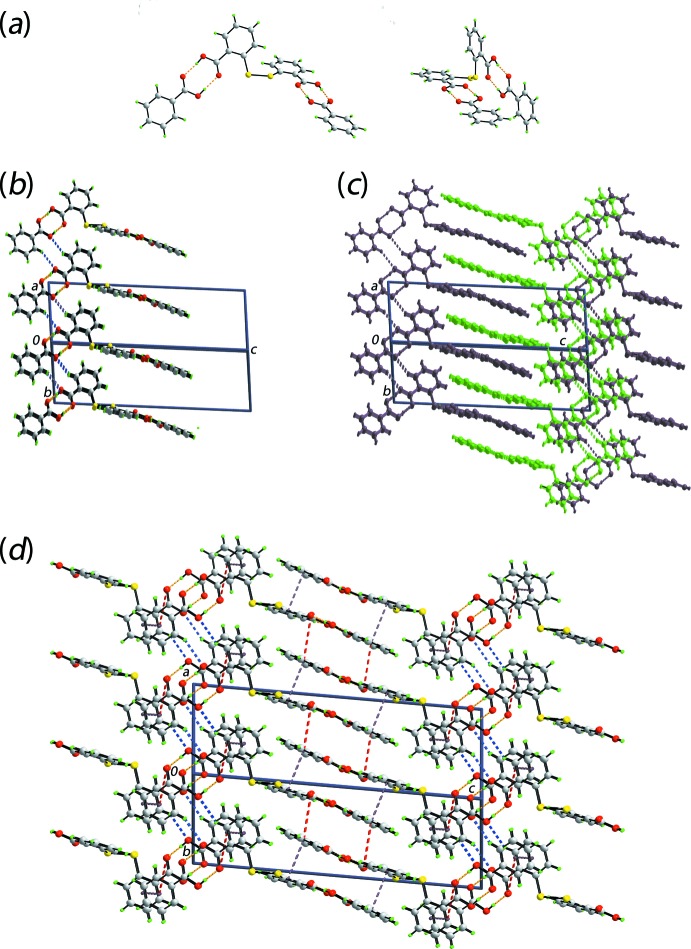
Mol­ecular packing in co-crystal (I): (*a*) two views of the three-mol­ecule aggregate with the the hy­droxy-O—H⋯O(carbon­yl) hydrogen bonds shown as orange dashed lines, (*b*) supra­molecular layer in the *ab* plane where the three-mol­ecule aggregates of (*a*) are linked by DTBA-C—H⋯O(hydroxy-BA), BA-C—H⋯O(hydroxy-DTBA) inter­actions, shown as blue dashed lines, (*c*) mutual orthogonal inter-digitation of symmetry-related layers and (*d*) a view of the unit-cell contents with π–π and C—O⋯π inter­actions shown as purple and red dashed lines, respectively.

The geometric parameters characterizing the inter­atomic contacts, as identified in *PLATON* (Spek, 2009 ▸), in the crystal of (I) as are given in Table 1 ▸. The mol­ecular packing of the crystal structure is mainly governed by hydrogen bonds formed between the carb­oxy­lic groups of DTBA and BA, whereby each terminus of the former connects via hy­droxy-O—H⋯O(hy­droxy) hydrogen bonds, leading to a non-symmetric, eight-membered {⋯HOC=O}_2_ homo-synthon as shown in the two views of Fig. 2 ▸(*a*). The resultant three-mol­ecule aggregates are connected through DTBA-C—H⋯O3(hydroxyl-BA) and BA-C—H⋯O1(hydroxyl-DTBA) inter­actions, to form non-symmetric, ten-membered {O⋯HCCC}_2_ homo-synthons leading to supra­molecular layers in the *ab* plane, Fig. 2 ▸(*b*). Owing to the nearly right-angle relationship between the rings in the DTBA mol­ecule, and the co-planarity between the carb­oxy­lic acid groups and the respective rings they are connected to, the layers also have a similar topology. Adjacent layers inter-digitate with other layers, on both sides, *i.e*. approximately orthogonally, as highlighted in Fig. 2 ▸(*c*). As illustrated in Fig. 2 ▸(*d*), the connections between layers are of two types and include π–π stacking inter­actions between DTBA and BA rings with the inter-centroid (C2–C7)⋯(C9–C14)^iv^ separation being 3.8093 (10) Å, an angle of inclination of 8.36 (8)° and an off-set of 1.40 Å for symmetry operation (iv): 1 − *x*, 1 − *y*, 1 − *z*. The second inter­action is a weak, parallel DTBA-hy­droxy-O1⋯π(C9–C14)^ii^ contact with a O1⋯ring centroid(C9–C14)^v^ separation of 3.9049 (14) Å and angle at O1 = 60.96 (9)° for symmetry operation (v): 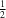 − *x*, 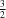 − *y*, 1 − *z*.

## Hirshfeld surface analysis and computational study   

To gain better understanding of the nature of the inter­molecular inter­actions identified in (I), the co-crystal and its individual components were subjected to a Hirshfeld surface analysis through the mapping of the normalized contact distance (*d*_norm_) as well as calculation of the inter­action energies using *CrystalExplorer* (Turner *et al.*, 2017 ▸) and in accord with a recent study (Tan & Tiekink, 2018 ▸). Briefly, the *d*_norm_ maps were obtained through the calculation of the inter­nal (*d*_i_) and external (*d*_e_) distances to the nearest nucleus (Spackman & Jayatilaka, 2009 ▸), while the inter­action energies were calculated using a dispersion-corrected CE-B3LYP/6-31G(d,p) quantum level of theory, as available in *CrystalExplorer* (Turner *et al.*, 2017 ▸). The total inter­molecular energy is the sum of energies of four main components, comprising electrostatic, polarization, dispersion and exchange-repulsion with scale factors of 1.057, 0.740, 0.871 and 0.618, respectively (Mackenzie *et al.*, 2017 ▸).

**Figure 3:**
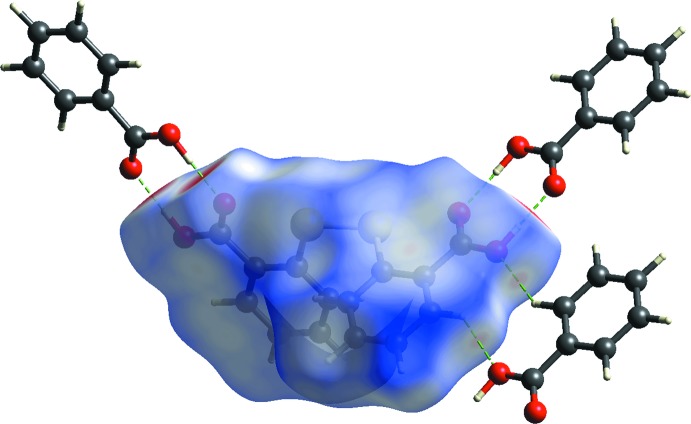
The Hirshfeld surface mapped with *d*_norm_ for the DTBA mol­ecule in (I) over the range −0.753 to 1.252 a.u., shown inter­acting with near-neighbour BA mol­ecules connected through hydrogen bonds (green dashed lines).

**Figure 4:**
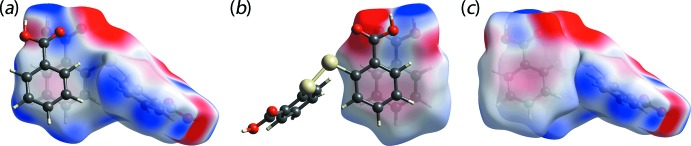
The electrostatic potential mapped over the Hirshfeld surface for (*a*) the DTBA mol­ecule, (*b*) the BA mol­ecule and (*c*) a BA mol­ecule involved in a π–π stacking inter­action with the ring of a DTBA mol­ecule. The isovalue was scaled between −0.026 to 0.056 a.u. for all surfaces.

The *d*_norm_ mapping of the three-mol­ecule aggregate is shown in Fig. 3 ▸. In general, the prominent hydrogen-bond inter­actions are readily identified from the intense red spots on the Hirshfeld surface which are dominated by the strong hy­droxy-O—H⋯O(carbon­yl) hydrogen bonds. The calculation of the relevant inter­action energies shows that it is the strongest among all of the specified contacts present in the crystal with the calculated (total) energy of −71.7 kJ mol^−1^, Table 2 ▸. By contrast, the diminutive red spots observed around the atoms involved in the benzene-C—H⋯O(hy­droxy) contacts, Table 1 ▸, are indicative of weak inter­actions, and this is confirmed through the calculated inter­action energy of merely −7.1 kJ mol^−1^. The short π–π inter­action involving the DTBA and BA benzene rings, mentioned in *Supra­molecular features*, has an inter­action energy of −21.7 kJ mol^−1^, *i.e*. more stable than the C—H⋯O inter­actions. The energy calculation reveals that such an inter­action is mainly dispersive in nature, *cf* Table 2 ▸, with the electrostatic character of the corresponding benzene rings being complementary, as demonstrated from the electrostatic surface mapped onto the Hirshfeld surfaces of the individual components of (I), Fig. 4 ▸(*a*) and (*b*), and the mol­ecular dimer sustained by π–π contacts in Fig. 4 ▸(*c*).

Other important but less significant contacts are noted through the Hirshfeld surface analysis such as a longer π–π inter­action between DTBA and BA rings with an inter-centroid (C2–C7)⋯(C9–C14)^v^ separation of 4.4323 (10) Å, an angle of inclination of 8.36 (8)° and an off-set of 2.74 Å for symmetry operation (v): 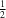 − *x*, 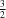 − *y*, 1 − *z*. In addition, a BA-benzene-C6—H⋯S contact (2.94 Å) is noted, Table 2 ▸.

**Figure 5:**
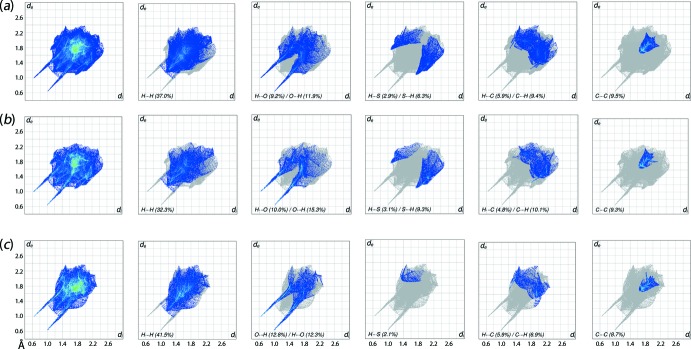
The full two-dimensional fingerprint plot and those delineated into H⋯H, O⋯H/H⋯O, S⋯H/H⋯S, C⋯H/H⋯C and C⋯C contacts for (*a*) (I), (*b*) DTBA and (*c*) BA, respectively.

A qu­anti­tative analysis of the Hirshfeld surfaces was performed through the generation of two-dimensional fingerprint plots by combining the *d*_i_ and *d*_e_ contact distances at the inter­val of 0.01 Å (McKinnon *et al.*, 2007 ▸). The overall fingerprint plot of the co-crystal (DTBA⋯BA) and the corresponding plots of the individual components are shown in Fig. 5 ▸ and percentage contributions are given in Table 3 ▸. In general, the overall bug-like fingerprint profiles of (I) and its individual components very much resemble to each other, as expected for DTBA and BA mol­ecules both with nearly identical donor–acceptor inter­actions. The major contribution to the overall Hirshfeld surfaces of (I) comprising H⋯H (37.0%; *d*_i_ + *d*_e_ ∼2.44 Å), O⋯H/H⋯O (21.1%; *d*_i_ + *d*_e_ ∼1.64 Å), S⋯H/H⋯S (9.2%; *d*_i_ + *d*_e_ ∼2.82 Å) and other contacts (8.0%). Among these contacts, only the O⋯H/ H⋯O, C⋯C and S⋯H/ H⋯S contacts are shorter than the respective sums of the van der Waals radii to result in meaningful inter­actions in the crystal, *i.e*. O⋯H, C⋯C and S⋯H = ∼1.72, ∼3.4 and ∼3.0 Å, respectively.

A close inspection on the corresponding decomposed fingerprint plots of the individual DTBA and BA mol­ecules reveals similar compositions as well as *d*_i_ + *d*_e_ distances except for the O⋯H/H⋯O inter­actions. Thus, overall (I) possesses 11.9% (inter­nal)-O⋯H-(external) and 9.2% of (inter­nal)-H⋯O-(external) close contacts as compared to that of 15.3 and 10.0%, respectively, for the individual DTBA mol­ecule, while the individual BA mol­ecule exhibits almost equivalent O⋯H and H⋯O contacts, *i.e*. 12.8 *versus* 12.3%. The apparent disparity arises as a result of the larger surface area to volume ratio for the DTBA mol­ecule as compared to the DTBA+BA aggregate when it is considered as a single entity, hence leading to greater exposure of the O⋯H/H⋯O contacts in DTBA within its surrounding inter­acting environment. A smaller disparity is evident for the (inter­nal)-S⋯H-(external)/(inter­nal)-H⋯S-(external) contacts, in that the former constitutes about 6.3% in (I) and 9.3% in DTBA, while the latter is about 2.9% in (I) and 3.1% in DTBA, respectively. The BA mol­ecule only exhibits (inter­nal)-H⋯S-(external) contacts that contribute about 2.1% to the overall Hirshfeld surface.

**Figure 6:**
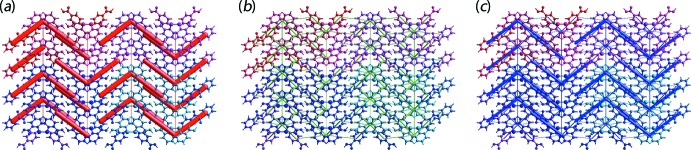
Energy framework of (I) as viewed down along the *a*-axis direction, showing the (*a*) electrostatic potential force, (*b*) dispersion force and (*c*) total energy diagrams. The cylindrical radii are proportional to the relative strength of the corresponding energies and they were adjusted to the same scale factor of 50 with a cut-off value of 5 kJ mol^−1^ within 4 × 4 × 4 unit cells.

In order to study the overall topology of the energy distributions in the crystal of (I), the energy framework was generated for a cluster of 4 × 4 × 4 unit cells using the same quantum level of theory as mentioned for the inter­action energy model. As shown in Fig. 6 ▸(*a*)–(*c*), the crystal is significantly governed by electrostatic force owing to the strong O—H⋯O inter­actions that result in an alternate V-shape energy topology across the *b*-axis direction. A relatively less significant, but essential dispersion contribution is also observed and arises from the *π*–*π* inter­actions spanning all benzene rings. Overall, it can be concluded that these inter­acting forces directed the assembly of the mol­ecules in (I).

## Database survey   

As mentioned in the *Structural commentary*, the DTBA mol­ecule is twisted about the central di­sulfide bond, having a C—S—S—C torsion angle of −83.19 (8)°. A survey of the literature indicates that this is a common feature of such mol­ecules. A search of the Cambridge Structural Database (Version 5.39; Groom *et al.*, 2016 ▸), indicates there are 33 different mol­ecules of DTBA. The C—S—S—C torsion angles span a range of approximately 20° with the narrowest angle of 80.06 (9)° found in the structure of a 1:1 co-crystal of DTBA with *trans*-1,2-bis­(4-pyrid­yl)ethene (Broker & Tiekink, 2007 ▸) and the widest angle of 100.98 (17)° was observed in in a co-crystal salt, *i.e*. [NH_4_][DTBA_H]DBTA (Murugavel *et al.*, 2001 ▸).

## Synthesis and crystallization   

All chemicals were of reagent grade and used as received without purification. 2-Thio­benzoic acid (Merck; 0.154 g, 0.001 mol) was mixed with benzoic acid (R&M; 0.122 g, 0.001 mol) and ground for 15 minutes in the presence of a few drops of methanol. The procedure was repeated three times. Colourless blocks were obtained by carefully layering toluene (1 ml) on an *N*,*N*-di­methyl­formamide (1 ml) solution of the ground mixture. M.p. 384.2–385.6 K. IR (cm^−1^): 3070 ν(C—H), 1677 ν(C=O), 1584 ν(C=C), 1415 δ(C—H), 706 δ(C=C), 684 δ(OCO).

## Refinement   

Crystal data, data collection and structure refinement details are summarized in Table 4 ▸. The carbon-bound H atoms were placed in calculated positions (C—H = 0.93 Å) and were included in the refinement in the riding-model approximation, with *U*_iso_(H) set to 1.2*U*_eq_(C). The oxygen-bound H atoms were located from difference-Fourier maps and refined without constraint.

## Supplementary Material

Crystal structure: contains datablock(s) global, I. DOI: 10.1107/S2056989018017097/hb7790sup1.cif

Structure factors: contains datablock(s) I. DOI: 10.1107/S2056989018017097/hb7790Isup2.hkl

Supporting information file. DOI: 10.1107/S2056989018017097/hb7790Isup3.cml

CCDC reference: 1882556

Additional supporting information:  crystallographic information; 3D view; checkCIF report

## Figures and Tables

**Table 1:** Hydrogen-bond geometry (Å, °)

*D*—H⋯*A*	*D*—H	H⋯*A*	*D*⋯*A*	*D*—H⋯*A*
O1—H1O⋯O4	0.88 (3)	1.74 (3)	2.6134 (17)	170 (2)
O3—H3O⋯O2	0.86 (3)	1.79 (3)	2.6535 (18)	178 (4)
C7—H7⋯O3^ii^	0.93	2.57	3.329 (2)	139
C14—H14⋯O1^iii^	0.93	2.59	3.395 (2)	145

Symmetry codes: (ii) 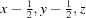; (iii) 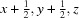.

**Table 2:** Inter­action energies (kJ mol^−1^) for selected close contacts

Contact	*E*_electrostatic_	*E*_polarization_	*E*_dispersion_	*E*_exchange-repulsion_	*E*_total_	Symmetry operation
O3—H3⋯O2/O1—H1⋯O4	−126.1	−29.0	−13.1	153.0	−71.7	1 − *x*, 1 − *y*, 1 − *z*
*Cg*1(C2–C7)⋯*Cg*2(C9–C14)	−0.1	−1.5	−41.8	25.5	−21.7	−*x*, − *y*, − *z*
*Cg*1(C2–C7)⋯*Cg*2(C9–C14)	−4.2	−1.3	−30.1	20.8	−18.7	− 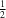 + *x*, 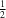 + *y*, *z*
C6—H6⋯S1	−10.2	−2.0	−13.5	15.8	−14.2	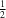 + *x*, 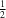 + *y*, *z*
C14—H14⋯O1/C7—H7⋯O3	−3.6	−0.9	−13.6	14.8	−7.1	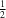 − *x*, 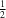 − *y*, 1 − *z*

**Table 3:** Percentage contributions of selected inter­atomic contacts to the Hirshfeld surface for (I) and for the the individual TDBA and BA mol­ecules

Contact	Percentage contribution		
	overall	TDBA	BA
H⋯H	37.0	32.3	41.5
O⋯H/H⋯O	21.1	25.3	25.1
S⋯H/H⋯S	9.2	12.4	2.1
C⋯H/H⋯C	15.3	14.9	12.8
C⋯C	9.5	9.3	8.7

**Table 4:** Experimental details

Crystal data	
Chemical formula	C_14_H_10_O_4_S_2_·2C_7_H_6_O_2_
*M*_r_	550.58
Crystal system, space group	Monoclinic, *C*2/*c*
Temperature (K)	100
*a*, *b*, *c* (Å)	8.2311 (1), 13.3220 (2), 22.7038 (3)
β (°)	95.864 (2)
*V* (Å^3^)	2476.55 (6)
*Z*	4
Radiation type	Cu *K*α
μ (mm^−1^)	2.41
Crystal size (mm)	0.20 × 0.11 × 0.06

Data collection	
Diffractometer	XtaLAB Synergy, Dualflex, AtlasS2
Absorption correction	Gaussian (*CrysAlis PRO*; Rigaku OD, 2018 ▸)
*T*_min_, *T*_max_	0.643, 1.000
No. of measured, independent and observed [*I* > 2σ(*I*)] reflections	32177, 2589, 2511
*R*_int_	0.039
(sin θ/λ)_max_ (Å^−1^)	0.630

Refinement	
*R*[*F* ^2^ > 2σ(*F* ^2^)], *wR*(*F* ^2^), *S*	0.039, 0.109, 1.08
No. of reflections	2589
No. of parameters	180
H-atom treatment	H atoms treated by a mixture of independent and constrained refinement
Δρ_max_, Δρ_min_ (e Å^−3^)	0.66, −0.49

Computer programs: *CrysAlis PRO* (Rigaku OD, 2018 ▸), *SHELXT* (Sheldrick, 2015*b* ▸), *SHELXL* (Sheldrick, 2015*a* ▸), *ORTEP-3 for Windows* (Farrugia, 2012 ▸), *OLEX2* (Dolomanov *et al.*, 2009 ▸), *Mercury* (Macrae *et al.*, 2006 ▸) and *publCIF* (Westrip, 2010 ▸).

## supplementary crystallographic information

### Crystal data

**Table:**

C_14_H_10_O_4_S_2_·2C_7_H_6_O_2_	*F*(000) = 1144
*M**_r_* = 550.58	*D*_x_ = 1.477 Mg m^−^^3^
Monoclinic, *C*2/*c*	Cu *K*α radiation, λ = 1.54184 Å
*a* = 8.2311 (1) Å	Cell parameters from 18092 reflections
*b* = 13.3220 (2) Å	θ = 3.9–76.2°
*c* = 22.7038 (3) Å	µ = 2.41 mm^−^^1^
β = 95.864 (2)°	*T* = 100 K
*V* = 2476.55 (6) Å^3^	Prism, colourless
*Z* = 4	0.20 × 0.11 × 0.06 mm

### Data collection

**Table:**

XtaLAB Synergy, Dualflex, AtlasS2 diffractometer	2589 independent reflections
Radiation source: micro-focus sealed X-ray tube, PhotonJet (Cu) X-ray Source	2511 reflections with *I* > 2σ(*I*)
Mirror monochromator	*R*_int_ = 0.039
Detector resolution: 5.2558 pixels mm^-1^	θ_max_ = 76.2°, θ_min_ = 3.9°
ω scans	*h* = −10→10
Absorption correction: gaussian (CrysAlis PRO; Rigaku OD, 2018)	*k* = −16→16
*T*_min_ = 0.643, *T*_max_ = 1.000	*l* = −28→28
32177 measured reflections	

### Refinement

**Table:**

Refinement on *F*^2^	Primary atom site location: dual
Least-squares matrix: full	Hydrogen site location: mixed
*R*[*F*^2^ > 2σ(*F*^2^)] = 0.039	H atoms treated by a mixture of independent and constrained refinement
*wR*(*F*^2^) = 0.109	*w* = 1/[σ^2^(*F*_o_^2^) + (0.0597*P*)^2^ + 4.0853*P*] where *P* = (*F*_o_^2^ + 2*F*_c_^2^)/3
*S* = 1.08	(Δ/σ)_max_ = 0.001
2589 reflections	Δρ_max_ = 0.66 e Å^−^^3^
180 parameters	Δρ_min_ = −0.49 e Å^−^^3^
0 restraints	

### Special details

**Table:**

Geometry. All esds (except the esd in the dihedral angle between two l.s. planes) are estimated using the full covariance matrix. The cell esds are taken into account individually in the estimation of esds in distances, angles and torsion angles; correlations between esds in cell parameters are only used when they are defined by crystal symmetry. An approximate (isotropic) treatment of cell esds is used for estimating esds involving l.s. planes.

### Fractional atomic coordinates and isotropic or equivalent isotropic displacement parameters (Å^2^)

**Table:**

	*x*	*y*	*z*	*U*_iso_*/*U*_eq_	
S1	0.50125 (5)	0.53534 (3)	0.29507 (2)	0.01870 (14)	
O1	0.29643 (16)	0.49404 (10)	0.46525 (5)	0.0239 (3)	
H1o	0.328 (3)	0.544 (2)	0.4890 (12)	0.042 (7)*	
O2	0.46078 (15)	0.57402 (9)	0.40809 (5)	0.0213 (3)	
O3	0.52518 (17)	0.72548 (10)	0.48274 (6)	0.0260 (3)	
H3o	0.502 (4)	0.676 (2)	0.4589 (13)	0.053 (8)*	
O4	0.35583 (16)	0.64485 (9)	0.53788 (5)	0.0237 (3)	
C1	0.3639 (2)	0.50608 (12)	0.41556 (7)	0.0179 (3)	
C2	0.3123 (2)	0.43148 (12)	0.36912 (7)	0.0175 (3)	
C3	0.3622 (2)	0.43865 (12)	0.31172 (7)	0.0172 (3)	
C4	0.3046 (2)	0.36819 (13)	0.26917 (7)	0.0192 (3)	
H4	0.336143	0.372512	0.231068	0.023*	
C5	0.2004 (2)	0.29140 (13)	0.28321 (8)	0.0213 (4)	
H5	0.162961	0.244879	0.254423	0.026*	
C6	0.1519 (2)	0.28379 (13)	0.33971 (8)	0.0220 (4)	
H6	0.083137	0.232083	0.349054	0.026*	
C7	0.2070 (2)	0.35389 (13)	0.38213 (8)	0.0208 (3)	
H7	0.173415	0.349359	0.419926	0.025*	
C8	0.4462 (2)	0.71641 (13)	0.53011 (7)	0.0195 (3)	
C9	0.4761 (2)	0.79789 (13)	0.57426 (7)	0.0196 (3)	
C10	0.4020 (2)	0.79189 (13)	0.62667 (7)	0.0212 (4)	
H10	0.331481	0.739174	0.632630	0.025*	
C11	0.4340 (2)	0.86479 (14)	0.66992 (8)	0.0234 (4)	
H11	0.384929	0.860972	0.704986	0.028*	
C12	0.5395 (2)	0.94372 (14)	0.66082 (8)	0.0238 (4)	
H12	0.561938	0.992032	0.690087	0.029*	
C13	0.6111 (2)	0.95056 (14)	0.60843 (8)	0.0247 (4)	
H13	0.680059	1.004025	0.602298	0.030*	
C14	0.5801 (2)	0.87766 (13)	0.56496 (8)	0.0225 (4)	
H14	0.628625	0.882052	0.529801	0.027*	

### Atomic displacement parameters (Å^2^)

**Table:**

	*U*^11^	*U*^22^	*U*^33^	*U*^12^	*U*^13^	*U*^23^
S1	0.0254 (2)	0.0140 (2)	0.0171 (2)	−0.00272 (14)	0.00424 (16)	−0.00104 (13)
O1	0.0350 (7)	0.0198 (6)	0.0178 (6)	−0.0079 (5)	0.0079 (5)	−0.0026 (5)
O2	0.0281 (6)	0.0181 (6)	0.0184 (6)	−0.0062 (5)	0.0054 (5)	−0.0024 (4)
O3	0.0380 (8)	0.0203 (6)	0.0212 (6)	−0.0079 (5)	0.0110 (5)	−0.0048 (5)
O4	0.0312 (7)	0.0193 (6)	0.0212 (6)	−0.0069 (5)	0.0062 (5)	−0.0025 (5)
C1	0.0218 (8)	0.0153 (8)	0.0169 (8)	0.0010 (6)	0.0027 (6)	0.0012 (6)
C2	0.0211 (8)	0.0132 (8)	0.0181 (8)	0.0005 (6)	0.0006 (6)	−0.0002 (6)
C3	0.0202 (8)	0.0120 (7)	0.0193 (8)	0.0003 (6)	0.0014 (6)	0.0006 (6)
C4	0.0221 (8)	0.0171 (8)	0.0184 (8)	−0.0002 (6)	0.0023 (6)	−0.0014 (6)
C5	0.0238 (8)	0.0159 (8)	0.0238 (8)	−0.0017 (6)	−0.0001 (6)	−0.0037 (6)
C6	0.0247 (8)	0.0147 (8)	0.0266 (9)	−0.0039 (7)	0.0022 (7)	0.0002 (6)
C7	0.0258 (8)	0.0169 (8)	0.0201 (8)	−0.0012 (7)	0.0033 (6)	0.0016 (6)
C8	0.0228 (8)	0.0173 (8)	0.0186 (8)	−0.0006 (6)	0.0027 (6)	0.0008 (6)
C9	0.0238 (8)	0.0163 (8)	0.0184 (8)	0.0008 (6)	0.0010 (6)	−0.0004 (6)
C10	0.0233 (8)	0.0186 (8)	0.0219 (8)	−0.0009 (6)	0.0030 (6)	0.0011 (6)
C11	0.0269 (9)	0.0235 (9)	0.0200 (8)	0.0015 (7)	0.0033 (7)	−0.0016 (7)
C12	0.0279 (9)	0.0197 (8)	0.0232 (9)	0.0015 (7)	−0.0006 (7)	−0.0048 (7)
C13	0.0276 (9)	0.0183 (8)	0.0282 (9)	−0.0035 (7)	0.0026 (7)	−0.0012 (7)
C14	0.0272 (9)	0.0198 (8)	0.0212 (8)	−0.0028 (7)	0.0051 (7)	−0.0008 (7)

### Geometric parameters (Å, º)

**Table:**

S1—S1^i^	2.0446 (8)	C6—H6	0.9300
S1—C3	1.7889 (17)	C6—C7	1.384 (2)
O1—H1o	0.88 (3)	C7—H7	0.9300
O1—C1	1.317 (2)	C8—C9	1.481 (2)
O2—C1	1.229 (2)	C9—C10	1.393 (2)
O3—H3o	0.87 (3)	C9—C14	1.395 (2)
O3—C8	1.318 (2)	C10—H10	0.9300
O4—C8	1.233 (2)	C10—C11	1.387 (2)
C1—C2	1.479 (2)	C11—H11	0.9300
C2—C3	1.409 (2)	C11—C12	1.393 (3)
C2—C7	1.400 (2)	C12—H12	0.9300
C3—C4	1.395 (2)	C12—C13	1.383 (3)
C4—H4	0.9300	C13—H13	0.9300
C4—C5	1.393 (2)	C13—C14	1.390 (3)
C5—H5	0.9300	C14—H14	0.9300
C5—C6	1.385 (2)		
			
C3—S1—S1^i^	105.68 (6)	C6—C7—H7	119.4
C1—O1—H1o	107.9 (18)	O3—C8—C9	115.08 (15)
C8—O3—H3o	110 (2)	O4—C8—O3	122.88 (16)
O1—C1—C2	114.38 (14)	O4—C8—C9	122.04 (15)
O2—C1—O1	122.96 (15)	C10—C9—C8	118.64 (16)
O2—C1—C2	122.65 (15)	C10—C9—C14	120.14 (16)
C3—C2—C1	121.58 (15)	C14—C9—C8	121.19 (15)
C7—C2—C1	119.01 (15)	C9—C10—H10	120.1
C7—C2—C3	119.38 (15)	C11—C10—C9	119.78 (17)
C2—C3—S1	119.84 (12)	C11—C10—H10	120.1
C4—C3—S1	121.18 (13)	C10—C11—H11	120.0
C4—C3—C2	118.96 (15)	C10—C11—C12	119.96 (17)
C3—C4—H4	119.7	C12—C11—H11	120.0
C5—C4—C3	120.63 (16)	C11—C12—H12	119.9
C5—C4—H4	119.7	C13—C12—C11	120.29 (17)
C4—C5—H5	119.7	C13—C12—H12	119.9
C6—C5—C4	120.52 (16)	C12—C13—H13	120.0
C6—C5—H5	119.7	C12—C13—C14	120.09 (17)
C5—C6—H6	120.3	C14—C13—H13	120.0
C7—C6—C5	119.36 (16)	C9—C14—H14	120.1
C7—C6—H6	120.3	C13—C14—C9	119.73 (16)
C2—C7—H7	119.4	C13—C14—H14	120.1
C6—C7—C2	121.15 (16)		

Symmetry code: (i) −*x*+1, *y*, −*z*+1/2.

### Hydrogen-bond geometry (Å, º)

**Table:**

*D*—H···*A*	*D*—H	H···*A*	*D*···*A*	*D*—H···*A*
O1—H1O···O4	0.88 (3)	1.74 (3)	2.6134 (17)	170 (2)
O3—H3O···O2	0.86 (3)	1.79 (3)	2.6535 (18)	178 (4)
C7—H7···O3^ii^	0.93	2.57	3.329 (2)	139
C14—H14···O1^iii^	0.93	2.59	3.395 (2)	145

Symmetry codes: (ii) *x*−1/2, *y*−1/2, *z*; (iii) *x*+1/2, *y*+1/2, *z*.
